# Quantification of Genes and Proteins Associated with Endothelial Cell Function After Different Exercise-Induced Shear Stress Intensities In Vitro

**DOI:** 10.3390/biology14091189

**Published:** 2025-09-03

**Authors:** Daniel Conde, Manuel Gomez, Alvaro N. Gurovich

**Affiliations:** 1 Clinical Applied Physiology (CAPh) Lab, The University of Texas at El Paso, El Paso, TX 79968, USA; mgomez26@utep.edu (M.G.); agurovich@utep.edu (A.N.G.); 2 Department of Physical Therapy and Movement Sciences, The University of Texas at El Paso, El Paso, TX 79968, USA; 3 Interdisciplinary Health Sciences Ph.D. Program, The University of Texas at El Paso, El Paso, TX 79968, USA

**Keywords:** exercise-induced shear stress, HUVEC, immunocytochemistry, Western blot, RT-PCR

## Abstract

Cell culture models are used in cardiovascular research to study molecular pathways associated with endothelial shear stress (ESS). However, previous studies have limited translation from in vivo ESS, especially across exercise intensities. Using the Ibidi pump system, ESS can be replicated in vitro to study exercise-induced changes in protein and gene expression. Currently, there are no standardized protocols describing how to translate exercise-induced ESS from in vivo data to in vitro models. This protocol addresses that gap by integrating human exercise data to generate physiologically relevant ESS from 18 to 60 dyn/cm^2^, replicating rest to high-intensity exercises. We describe the use of the Ibidi pump system to expose human umbilical vein endothelial cells (HUVEC) to exercise-induced ESS from in vivo data, followed by protein (Western blot and immunocytochemistry) and gene (reverse transcription polymerase chain reaction) analysis. The steps include ESS determination at different exercise intensities, culturing HUVEC, Ibidi pump system setup, and molecular analysis. Even though the sample experiment uses a low ESS, representative of low-intensity exercise, this model can be adapted to higher exercise intensities. Potential limitations and solutions are also discussed.

## Simple Summary

This protocol describes how to simulate different exercise-induced endothelial shear stress (ESS) intensities in vitro using data obtained during real human exercise. We show how to collect exercise data including VO_2_ max, lactate threshold, and arterial blood flow patterns, and then use this information to program a perfusion system to reproduce the same ESS in cultured endothelial cells. By controlling the ESS in the laboratory, researchers can study how exercise affects the health and function of blood vessels at a cellular and molecular level. This can help in the identification of mechanisms by which exercise protects the heart and blood vessels and prevents and improves the treatment for cardiovascular diseases; this can also help assess the effects of new drugs in an environment that replicates exercise conditions. Our methods simulate forces from rest to high-intensity exercise and can be applied to the study of cardiovascular and vascular biology physiology.

## 1. Introduction

The vascular endothelium is the first layer of defense in preventing vascular disease [1]. Initially considered an inactive barrier, the endothelium is now considered a vital organ responsible for maintaining vascular health. The function of key molecules is vital to maintain vascular homeostasis and tone and to prevent endothelial dysfunction [2,3]. Some of these molecules are the vasodilator gas nitric oxide (NO), which is produced by the dimeric enzyme endothelial nitric oxide synthase (eNOS); sirtuin 1, a protein involved in the regulation of endothelial oxidative stress; and endothelin-1, a vasoconstrictor and proinflammatory peptide upregulated in dysfunctional endothelium [4,5,6]. The expression of these peptides and their associated genes is mediated by the endothelial shear stress (ESS) intensity created by blood flow [7]. However, there is a lack of a standardized in vitro model using exercise-induced values directly measured in vivo, rather than arbitrary or fixed low shear stress intensities. Addressing this limitation could improve the translational value of vascular cell culture studies by better mimicking the physiological stimuli observed in different exercise intensities.

Different in vitro models have been used to study endothelial cell function using human umbilical endothelial cells (HUVEC) [8]. Although these studies describe the use of different perfusion systems to create ESS, they do not aim to reproduce the high ESS created during exercise [9,10]. This study specifically addresses that gap by describing the complete workflow, from the determination of VO_2_ max and lactate threshold to the calculation of shear stress and subsequent in vitro application, paired with detailed methods for gene and protein expression analysis. When simulating exercise-induced ESS, it is crucial to produce a shear force that mimics those produced during exercise. Recently, our group developed an in vitro exercise model to study the changes in endothelial function markers by applying exercise-induced ESS [11,12]. We used different exercise-induced ESS intensities in vitro that corresponded to those produced by different exercise intensities in vivo [13,14].

The methods we present here are ideal for assessing intensity-specific exercise-induced ESS in vivo, applying those intensities in an in vitro model, and assessing protein and gene expression of HUVEC subjected to exercise-induced ESS. Although our methods describe specific ESS intensity, targets, controls, and cell types, they can be easily modified to simulate other conditions. While in vivo studies are needed for understanding systemic responses, in vitro ESS models provide a powerful complementary approach to study cellular and molecular mechanisms in isolation. This allows assessment of how specific ESS intensities affect endothelial cells, testing the hypothesis before clinical trials, and potentially tailoring exercise prescriptions and therapeutic interventions.

Our methods present several advantages over those previously described. Most notably, our methods allow for an ESS up to 60 dyne (dyn; 1 dyn = 10^−5^ N)/cm^2^ to simulate high-intensity exercise-induced ESS, while other assays use ESS of only 25 dyn/cm^2^, which represents resting conditions [11,15,16]. Also, our methods can be paired with other established protein and gene extraction and other detection methods not presented here, including but not limited to enzyme-linked immunosorbent assay, flow cytometry, and Northern and Southern blots. Another significant advantage of our methods is that all materials are available from commercial vendors, and no special preparation of reagents is needed.

This lab protocol shows the feasibility of studying proteins and genes associated with endothelial cell function under exercise-induced ESS in vitro. We first determine intensity-specific exercise-induced ESS, seed and subculture HUVEC in special slides used by the Ibidi pump system, set up the pump system to simulate exercise-induced ESS conditions, and assess protein and gene expression using established detection methods.

## 2. Materials and Methods

General equipment, materials, and consumables required are listed below. Specific materials and equipment from recommended brands are listed in Table 1 and Table 2.

- Cycle ergometer;
- Scale with a stadiometer;
- Sphygmomanometer or automated blood pressure monitor;
- Capillary tubes;
- Capillary tube sealing compound;
- Ultrasound with a 12 to 18 Hz transducer;
- Pulsed-Wave Doppler;
- T-75 flasks;
- Biosafety cabinet;
- 70% ethanol;
- Water bath at 37 °C;
- Serological pipette;
- 10 mL serological pipette tips;
- Inverted microscope;
- Incubator (37 °C and 5% CO_2_);
- Hank’s balanced salt solution (HBSS);
- Dulbecco’s phosphate-buffered saline (DPBS);
- Trypsin ethylenediaminetetraacetic acid (EDTA);
- Trypsin neutralizing solution (TNS);
- 50 mL conical tubes;
- Centrifuge for 50 mL tubes;
- Hemocytometer or cell counter;
- Plastic hose clips;
- Syringe coupler;
- Paraformaldehyde;
- Triton X-100;
- Bovine serum albumin (BSA);
- 4′,6-diamino-2-phenylindole (DAPI);
- Confocal microscope;
- 1 mL syringe;
- 20-gauge syringe;
- 1.5 microcentrifuge tubes;
- Countertop centrifuge with temperature regulation;
- Nanodrop spectrophotometer;
- PCR plate;
- Thermocycler;
- Phenylmethylsulphonyl fluoride (PMSF);
- 25-gauge syringe;
- Bicinchoninic acid assay;
- Dithiothreitol (DTT);
- Tris glycine sodium dodecyl sulfate (Tris, glycine, SDS) buffer;
- Western blot incubation boxes;
- Plate shaker;
- Tris buffered saline (TBS);
- Micropipettes with corresponding pipette tips.

### 2.1. Determination of Exercise Intensities for Exercise-Induced Endothelial Shear Stress In Vivo

The determination of exercise intensities and corresponding ESS intensities followed our previously published protocols [10,11,14], describing maximal oxygen uptake (VO_2_ max) testing, lactate threshold determination, Doppler ultrasound acquisition, and ESS calculation in detail. Only key steps are summarized here to maintain reproducibility and avoid redundancy.

1. Measure the participants’ height and weight using a standard weighing scale and stadiometer.
2. Adjust the height of the seat to allow knee flexion between 5° and 15°.
3. Have the participant sit quietly on the cycle ergometer for 10 min to ensure sympathetic activity does not affect the blood pressure readings.
  - Measure the participant’s blood pressure twice at the end of the 10 min and repeat the measurement three times.
  - Average blood pressure measurements to establish a baseline.
4. Perform a VO_2_ max test following the American Heart Association (AHA) and the American College of Sports Medicine (ACSM) guidelines [17,18].
  - Participants must complete 2-min stages until volitional exhaustion.
  - Increase the workload by 25 Watts after each stage.
5. Measure VO_2_, heart rate (HR), and La at the end of each 2-min stage.
  - VO_2_ is measured from the inspired and expired air of the participant connected to the metabolic cart.
  - HR is measured with an H10 heart rate sensor placed on the chest.
  - La is measured using a micro-sample from the earlobe with the Lactate Plus Meter.
6. At the end of the test, determine three exercise intensities by determining 3 La curves as follows:
  - Low-intensity—0–2 mmol/L.
  - Moderate-intensity—2–4 mmol/L.
  - High-intensity—>4 mmol/L.

### 2.2. Determination of Exercise-Induced Shear Stress

**Figure 1:**
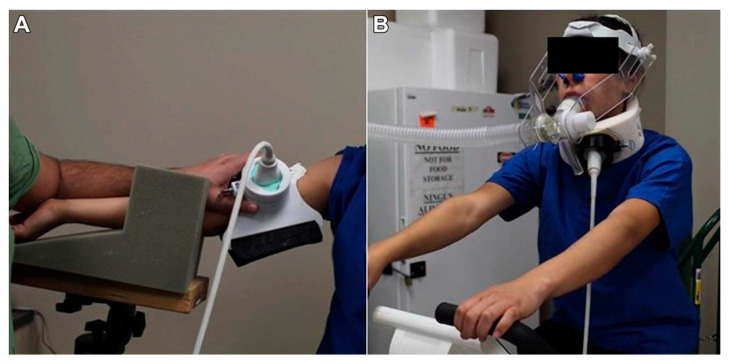
Blood flow pattern measurements using ultrasound imaging on the brachial (**A**) and carotid (**B**) arteries. Adapted from Gurovich et al., 2021 [10].

1. Determine the participant’s hematocrit from two earlobe capillary blood samples.
  - Collect the blood using hematocrit capillary tubes and seal them.
  - Centrifuge and determine hematocrit using a semi-automated hematocrit measuring device.
2. Have the participant exercise for 5 min at each predetermined intensity.
  - Monitor VO_2_ and HR throughout the exercise session.
  - Assess La at min 2 and 4 of each exercise intensity to confirm that the exercise intensity corresponds to the La expected at each intensity.
3. Measure blood flow patterns in the carotid artery and/or brachial artery using the 12 to 18 Hz ultrasound transducer.
  - Brachial artery—5 cm proximal to the antecubital fossa. Arm placed on a flat surface, maintain ~80° of shoulder abduction and a 35° to 45° flexion (Figure 1A).
  - Common carotid—lateral aspect of the neck (Figure 1B).
4. Adjust the ultrasound settings to a frequency of 12 Hz and 3 cm depth for the brachial artery and 18 Hz and 3–5 cm depth for the common carotid artery.
5. Identify a transverse section of the artery and center it.
6. Rotate the transducer 90° to obtain a longitudinal view of the artery and use a Pulsed-Wave Doppler to record blood flow velocity.
  - Ensure an insonation angle < 60°.
  - For the carotid artery, place the Doppler 5 to 10 mm distal to the bifurcation.
  - For the internal and external carotid arteries, move the Doppler 5 to 12 mm proximal to the bifurcation.
7. The ESS calculation follows the Womersley approximation, where the shear rate (SR) is determined from the measured artery diameter and peak systolic velocity obtained via Doppler ultrasound, and blood viscosity is estimated from measured hematocrit values. All parameters are recorded at each exercise intensity to ensure accuracy in replicating the corresponding shear stress in vitro. The ESS is then calculated as follows:
  - ESS = μ · SR.
    SR = 2K · V/D.
    - μ is blood viscosity.
    - SR is the shear rate.
    - V is the peak systolic velocity.
    - D is the artery diameter.
    - K is the complex factor dependent on the Womersley parameter α.
  - A = (D/2) · (ω/[μ/ρ])^1/2^.
    - D is the artery diameter.
    - ω is the angular frequency of flow pulsation (ω = freq · 2π).
    - ρ is blood density.
    - μ is blood viscosity.

### 2.3. HUVEC Seeding

1. Place the MesoEndo cell growth medium and the frozen vial of HUVEC in the water bath and monitor the cells closely.
  - Once there is minimal ice, remove the cells and the media, disinfect them with 70% ethanol to sterilize the exterior surfaces before introducing them into the biosafety cabinet, following standard aseptic technique guidelines [19,20], and place them in the biosafety cabinet.
  - Pipette 20 mL of media into a T-75 flask.
  - Resuspend the cells inside the vial by slowly pipetting up and down five times and transfer the cells to the T-75 flask containing the growth media.
  - Gently rock the flask for 10 s to distribute the cells evenly in the flask.
    - Check cell viability and distribution with an inverted microscope.
  - Place the cells in the incubator overnight.
  - After overnight incubation, check for cell adhesion and growth, and change the media to remove traces of dimethyl sulfoxide used during cryopreservation.
  - Change cell media every other day until cells reach 80% confluence.
    - Confluence is evaluated visually using the inverted microscope, estimating the proportion of growth surface covered by cells, consistent with standard cell culture guidelines [20].
    - Overconfluent cells become senescent.

### 2.4. HUVEC Subculture into Ibidi μ-Slide I Luer

**Figure 2:**
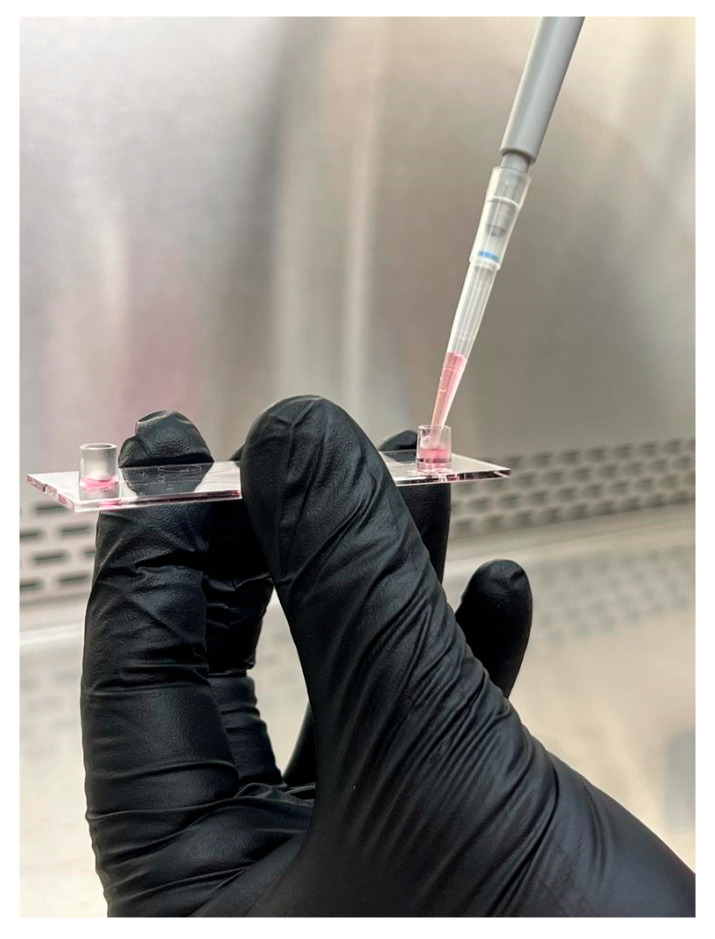
The HUVEC suspension is slowly added to the slide column to avoid bubble formation.

1. Place the perfusion set, μ-slides, and media inside the incubator overnight to avoid bubble formation inside the slides.
2. Remove the media from the T-75 containing the HUVEC inside the biosafety cabinet and wash them twice with HBSS or DPBS (Cell Applications, San Diego, CA, USA).
3. After the second wash, add 5 mL of trypsin EDTA (Cell Applications, San Diego, CA, USA), rock the flask to cover the cells completely, and immediately remove 4.5 mL of trypsin EDTA.
4. Monitor trypsinization under the microscope.
  - Once the cells become round, tap the flask lightly to detach them from the flask.
5. Add 5 mL of TNS (Cell Applications, San Diego, CA, USA) and transfer the cell suspension to a 50 mL conical tube.
  - Rinse the flask with 5 mL of TNS and transfer it to the 50 mL conical tube containing the cell suspension.
6. Centrifuge the conical tube for 5 min at 220× *g* to pellet the cells.
7. Place the 50 mL conical tube inside the biosafety cabinet and remove the supernatant without disturbing the pellet.
8. Loosen the pellet by flicking the tip of the tube and add 2 mL of growth media.
  - Resuspend the cells by slowly pipetting up and down.
9. Count the cells and make a cell suspension with a density of 1 × 10^6^ cells/mL
  - Dilute the cells with growth media if necessary.
10. Add 150 μL of cell suspension into the channel inlet of the Ibidi μ-slide (Figure 2).
  - Add the cell suspension slowly to avoid bubble formation inside the slide.
11. Incubate the cells until they reach 80% confluence
  - Change the media every other day.

### 2.5. Exercise-Induced ESS Experiments Using the Ibidi Pump System

**Figure 3:**
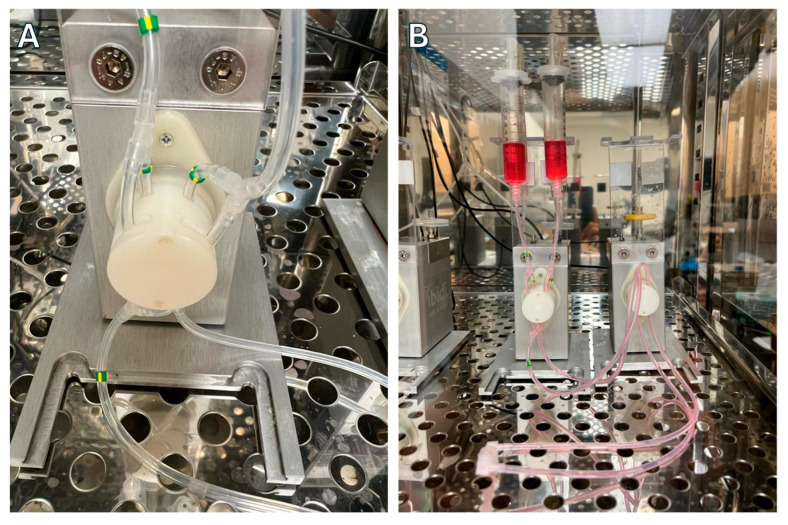
Fluidic unit setup. The perfusion set is connected to the fluidic unit used for unidirectional flow, with the yellow/green ends placed in the posterior pinch valves (**A**). The perfusion set is also connected to the fluidic unit used to create the pulses (**B**).

**Figure 4:**
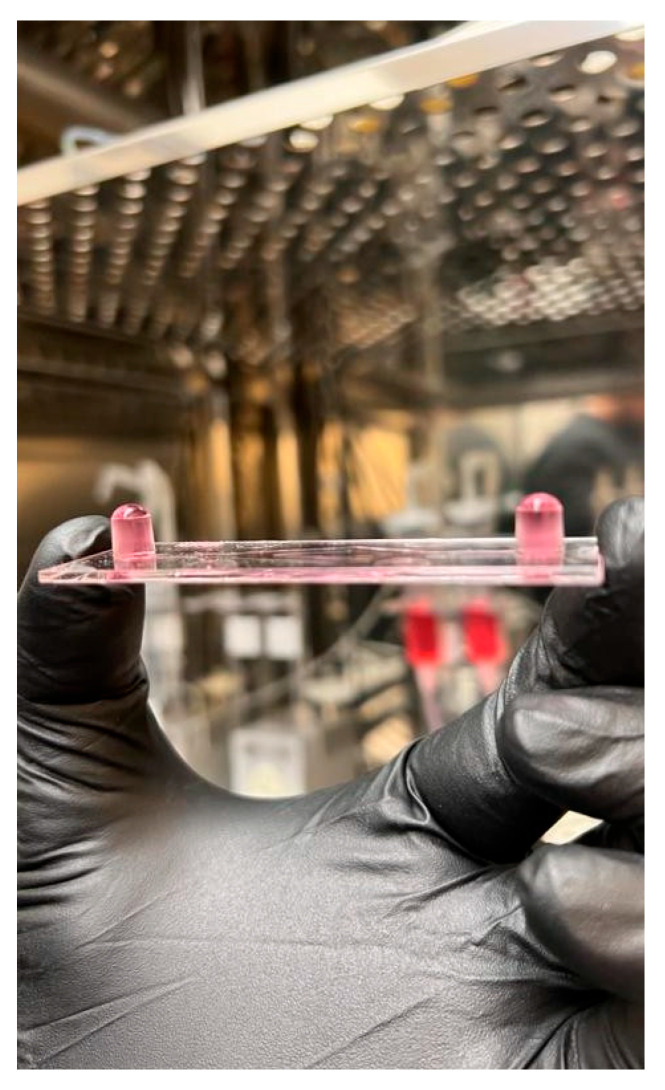
Slide showing overflowing media before connecting to the perfusion set. The overflowing media prevents the formation of bubbles when connecting to the perfusion set.

1. Place two fluidic units inside the incubator and attach the perfusion set as shown in Figure 3A,B.
2. Pinch the line with a plastic hose clip, add 5 mL of media to each syringe, and connect the two ends of the perfusion set with the syringe coupler.
  - Once the two ends are connected, remove the hose clip.
3. Using the Ibidi Pump Flow Control Software version 1.6.2, set the following parameters to remove any bubbles in the line.
  - Under the fluidic unit set up tab, select the yellow/green perfusion set without any slide.
  - Click Apply New Settings.
  - Under the flow parameters tab, set the shear stress to 80 dyn/cm^2^.
  - Click the “play” button and watch the bubbles in the line. Once the line is free of bubbles, click on the stop button.
4. With the line free of bubbles, pinch the line, remove the plastic connector, add approximately 50 μL of media to one of the slide’s channel inlets, and connect to the perfusion set (Figure 4).
5. Set the shear stress to the appropriate value calculated from the in vivo measurements.
6. Set the fluidic unit 1 (P1) as unidirectional and the fluidic unit 2 (P2) as oscillatory.
7. Dynamic shear stress settings are programmed to match the pulsatile nature of in vivo blood flow.
  - For this example, a frequency of 60 pulses per minute (PPM) was set with an oscillatory interval of 0.5 s (1 Hz).
  - Higher or lower PPM values can be calculated using the following formula:
    Oscillatory interval (s) = 60/PPM.
  - The cycle duration is set to match the total planned exposure time. For example, 6 h for the presented experiments.
    - The system will automatically stop at the end of the cycle.
  - If two slides run simultaneously, set P3 as unidirectional and P4 as oscillatory.

### 2.6. Protein Detection Using Immunocytochemistry (ICC)

1. Remove the cell media from the slide and wash it with 150 μL of DPBS three times.
2. Remove the DPBS and fix the cells by adding 150 μL of 2% paraformaldehyde (Fisher Scientific, Hampton, NH, USA), and incubating at room temperature for 10 min.
3. Remove the paraformaldehyde and wash the cells three times with DPBS.
4. After the last wash, add 150 μL of 0.1% Triton X-100 (Fisher Scientific, Hampton, NH, USA) and incubate at room temperature for 10 min.
5. Remove the Triton X-100 and wash the cells twice with DPBS.
6. Remove the DPBS and add 150 μL of 1% BSA (Sigma-Aldrich, St Louis, MO, USA) and incubate overnight at 4 °C
7. After incubation, perform the following steps in a dark room.
  - Add 150 μL of primary antibody (eNOSp; Abcam, Waltham, MA, USA) diluted 1:100 in 1% BSA and incubate for 60 min at 37 °C.
  - Remove the primary antibody and wash the cells three times with DPBS.
  - Add 150 μL of secondary antibody (Alexa Fluor 488; Invitrogen, Waltham, MA, USA) diluted 1:1000 in 1% BSA and incubate for 60 min.
  - Remove the secondary antibody and wash the cells three times with DPBS.
  - Add 150 μL of DAPI (Fisher Scientific, Hampton, NH, USA) diluted 1:1000 in DPBS and incubate for 5 min.
  - Remove the DAPI and wash the cells three times with DPBS.
  - Add 150 μL of fresh DPBS and visualize the cells using a confocal microscope (LSM 700, Carl Zeiss Microscopy GmbH, Jena, Germany) or store at 4 °C protected from light.
    - Visualize within a week to prevent degradation of the dyes.

### 2.7. mRNA Extraction and qRT-PCR

1. Remove the cell media, wash the cells five times with DPBS.
2. Add 100 μL of RNA lysis buffer (buffer RLT) from the Qiagen RNeasy Micro Kit to lyse the cells.
3. Attach a 1 mL syringe to one of the channel inlets and move the plunger quickly up and down several times to shear out the lysed cells.
  - Place the cell lysate in a 1.5 mL microcentrifuge tube.
4. Add another 100 μL of buffer RLT from the Qiagen RNeasy Micro Kit to the slide and shear out any remaining cells.
  - Place the cell lysate in the same 1.5 mL microcentrifuge tube.
5. Mix the cell lysate with an additional 150 μL of buffer RLT to have a total volume of 350 μL.
6. Attach a 20-gauge needle to the syringe and pass the entire cell lysate 15 times.
  - At this point, the cell lysate can be stored at −20 °C for up to one week.
7. Add 350 μL of 70% ethanol (Fisher Scientific, Hampton, NH, USA), mix by pipetting up and down, and transfer it to a MinElute spin column.
8. Centrifuge the column at 8000× *g* for 15 s and discard the flow-through in the collection tube.
  - Repeat this step for any remaining lysate.
9. Add 350 μL of buffer RW1 from the Qiagen RNeasy Micro Kit to the column, centrifuge at 8000× *g* for 15 s, and discard the flow-through and collection tube.
10. Insert the column into a new collection tube and add 500 μL of buffer RPE from the Qiagen RNeasy Micro kit, centrifuge at 8000× *g* for 15 s, and discard the flow through.
  - Repeat this step once.
11. Add 500 μL of 80% ethanol (Fisher Scientific, Hampton, NH, USA), centrifuge at 8000× *g* for 2 min, and discard the flow-through.
  - Completely dry the column by centrifuging the column at >12,000× *g* for 5 min.
  - Discard the flow-through and collection tube.
12. Place the column in a 1.5 mL microcentrifuge tube, elute the RNA by adding 14 μL of RNase-free water from the Qiagen RNeasy Micro Kit, incubate for 3 min, and centrifuge at >12,000× *g* for one min.
  - The eluted RNA can be stored at −20 °C for up to a month.
  - Measure the RNA concentration using a nanodrop spectrophotometer (Nanodrop One, Fisher Scientific, Hampton, NH, USA) at 260 nm.
  - The 260/280 ratio provides the purity of the eluted RNA.
    - ≥1.8 and ≤2.0 indicate a pure RNA sample.
13. Prepare a reverse transcription master mix for up to 2 μg of mRNA using the high-capacity cDNA reverse transcription kit (Thermo Scientific, Waltham, MA, USA), following the volumes in Table 3.
14. Load 10 μL of the reverse transcription master mix in a PCR plate and 10 μL of the eluted mRNA and load the plates/tubes in a thermocycler following the settings of Table 4.
15. Take the samples from the thermocycler (GeneAmp PCR system 9700, Applied Biosystems, Waltham, MA, USA) and prepare a PCR master mix following the volumes in Table 5.
16. Load 18 μL of the PCR master mix in a PCR plate and 2 μL of cDNA, seal the plate using adhesive PCR plate seals.
17. Determine gene expression using the StepOne Plus PCR system (or similar instrument) using TaqMan fast settings in Table 6.
18. Determine gene expression using a relative standard curve, comparative threshold cycle, or other methods [21].

### 2.8. Protein Extraction and Western Blot Analysis

1. Place 10 mL DPBS, 1 mL cell lysis buffer with 1 mM PMSF (Thermo Scientific, Waltham, MA, USA), two 1.5 mL microcentrifuge tube, 1 mL syringe, 25-gauge syringe, and microcentrifuge tube on ice.
2. Remove four Ibidi slides and place them on ice.
  - All the extraction steps must be performed on ice to prevent protein degradation.
3. Wash the slides three times with 100 μL of DPBS.
  - After the third wash, do not remove the DPBS until ready to lyse the cells.
4. Remove the DPBS from the slide, add 200 μL of cold Cell Signaling’s cell lysis buffer with PMSF, and incubate for 5 min.
  - Use the syringe to remove any bubbles that may form inside the slide.
5. Connect the syringe to one of the slide’s columns and shear out the cells.
6. Remove the DPBS from the next slide, add the lysed cells inside the syringe, and repeat steps 4 and 5.
7. Once the contents from all four slides are collected, place the lysed cells in the 1.5 mL microcentrifuge tube.
8. Attach the 20-gauge needle to the syringe and pass the entire cell lysate 10 times.
  - Pass the lysate slowly to avoid excessive bubble formation.
9. Centrifuge the lysate at 14,000× *g*, 4 °C for 10 min to pellet the cells.
10. Collect the supernatant in the remaining microcentrifuge tube and proceed to assess the protein concentration or freeze the sample.
  - Use a BCA assay or other protein concentration assay.
  - The sample can be stored at −20 °C for up to one week.
11. Prepare the Mini-PROTEAN electrophoresis (Biorad, Hercules, CA, USA) system.
  - Place the precast gel into the cassette.
  - Add tris, glycine, SDS buffer (Fisher Scientific, Hampton, NH, USA) accordingly.
12. Dilute the protein sample to load 30 μg in each gel well.
  - Use Licor’s 4X protein sample loading buffer with 1M DTT.
13. Run the electrophoresis system at 150 V for 5 min to clean the precast gel from impurities.
14. Load 2 μL of Biorad’s precision plus protein dual color standard in the first well and 30 μL of protein sample in the remaining wells.
15. Run the electrophoresis system at 50 V for about 2.5 h, until the proteins separate and reach the bottom of the gel.
16. Stop the current, remove and disassemble the cassette, rinse the gel, and place it in the transfer pack.
17. Place the transfer pack in the trans-blot turbo system and transfer the proteins to the PVDF membrane.
  - Use the presets “turbo” and “1 mini TGX”
18. Block the membrane in a black incubation box, using the Licor’s intercept (TBS) blocking buffer, at room temperature for 2 h on a shaker set at 130 revolutions per minute (RPM).
  - The membrane must be completely covered by blocking buffer and free of bubbles.
19. After blocking, remove the blocking buffer and incubate the membrane in primary antibody overnight at 4 °C on a shaker (Pro 30 Reciprocal Laboratory Shaker, Labnet, Edison, NJ, USA) set at 130 RPM.
  - eNOS recommended dilution is 1:1000 for Western blot.
  - The primary antibody is prepared with blocking buffer.
20. After incubation with primary antibody, wash the membrane three times using TBS (Fisher Scientific, Hampton, NH, USA).
21. After washing, incubate the membrane in Licor’s IRDye 800 fluorescent secondary antibody at room temperature for 2 h, protected from light.
  - The recommended dilution for IRDye 800 is 1:15,000.
22. After incubation with secondary antibody, wash the membrane three times with TBS and scan the membrane using a near-infrared fluorescent scanner (Odyssey DLx, Licor, Lincon, LE, USA).
  - The membrane can be stored in TBS at 4 °C for two weeks or dried for long-term storage.
23. Repeat steps 19–22 for incubation with loading control antibody.

## 3. Results

The following representative results are from two experiments following the protocols previously described. Both experiments used HUVEC exposed to two different ESS intensities: resting ESS at 18 dyn/cm^2^ and 60 PPM, and low-intensity ESS at 35 dyn/cm^2^ and 100 PPM. After exposure to the exercise-induced ESS, target proteins phosphorylated nitric oxide synthase (eNOSp) and actin were stained using ICC techniques [22], gene expression of *NOS3* gene using RT-qPCR, and protein expression of nitric oxide synthase (eNOS) using Western blot [12].

### 3.1. Protein Detection Using ICC

**Figure 5:**
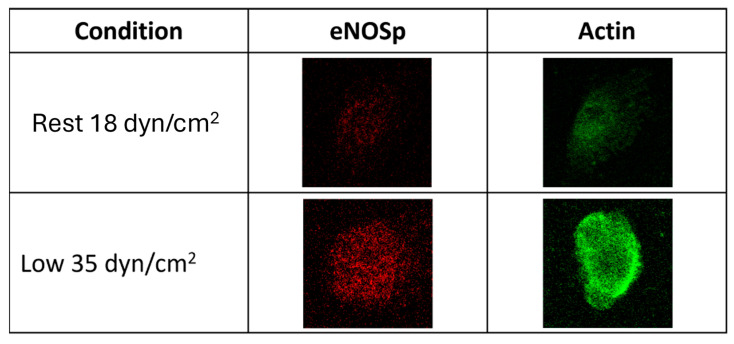
Representative images of one ROI of an Ibidi slide. Resting ESS (18 dyn/cm^2^, 60 PPM) and low-intensity ESS (35 dyn/cm^2^, 100 PPM) are shown.

The analysis of 12 regions of interest (ROI) from ICC images resulted in higher expression of eNOSp and actin after exposure to low-intensity ESS for six hours when compared to resting ESS (Figure 5).

### 3.2. Gene Expression Analysis

**Figure 6:**
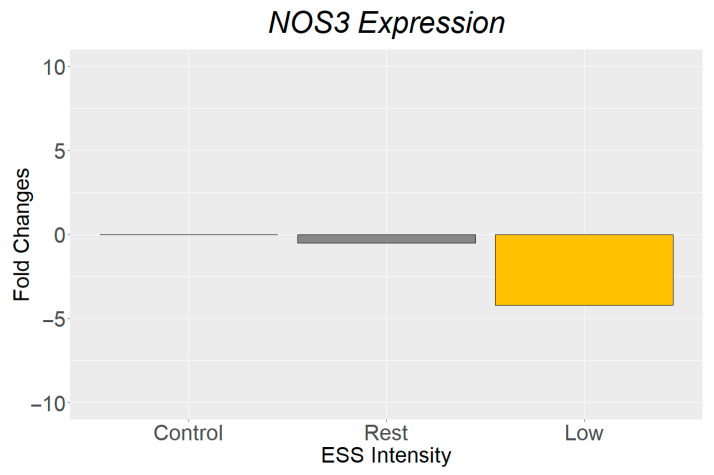
NOS3 gene expression fold changes relative to GAPDH after exposure to resting ESS (18 dyn/cm^2^, 60 PPM) and low-intensity ESS (35 dyn/cm^2^, 100 PPM).

The analysis of changes in *NOS3* expression, relative to the housekeeping gene Glyceroldehyde-3-Phosphate Dehydrogenase (*GAPDH*), was calculated using the 2^−ΔΔcT^ method. The results show a downregulation in *NOS3* expression of HUVEC exposed to resting ESS and a further reduction with low-intensity ESS, compared to unstimulated control (Figure 6).

### 3.3. Protein Expression

**Figure 7:**
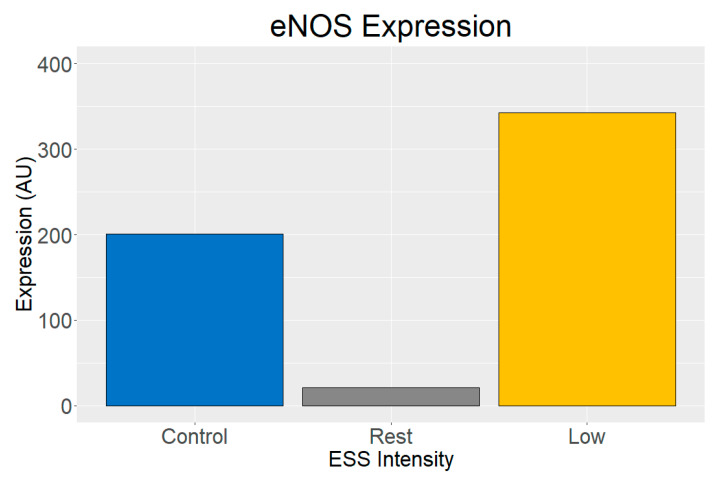
Normalized eNOS expression in HUVEC exposed to resting ESS (18 dyn/cm^2^, 60 PPM) and low-intensity ESS (35 dyn/cm^2^). Values are expressed in arbitrary units (AU).

The fluorescence intensity of the immunoblots was quantified as arbitrary units (AU) to assess the changes in eNOS after exposure to resting and low-intensity ESS. The fluorescence intensity of eNOS was normalized to the loading control GAPDH. The results show a decrease in eNOS after exposure to resting ESS and an increase after exposure to low-intensity ESS (Figure 7). The uncropped blot images are available as Supplemental Materials Figures S1–S3.

## 4. Discussion

This study presents a novel and adaptable protocol for an in vitro model of exercise-induced ESS. It integrates in vivo-derived hemodynamic data with a commercially available perfusion pump system. The successful exposure of HUVEC to resting and low-intensity ESS demonstrated that specific cellular responses can be replicated in a controlled environment. The ability to mimic physiological ESS levels corresponding to different exercise intensities enhances the translational relevance of this model, especially when compared to previous models that often apply sub-physiological ESS levels. The capacity to replicate ESS intensities across the full physiological spectrum, from rest (18 dyn/cm^2^) to high-intensity exercise (60 dyn/cm^2^), is the key, novel feature compared to earlier studies limited to static or low shear stress conditions.

The ICC and Western blot findings showed increased eNOSp expression and actin cytoskeletal alignment after exposure to low-intensity ESS. These findings align with the literature showing that ESS acts as a strong modulator of endothelial nitric oxide synthase activity, promoting vascular homeostasis. Interestingly, gene expression analysis showed a paradoxical reduction in *NOS3* expression even at low-intensity ESS. This may suggest a previously unexplored role of exercise-induced ESS as a promoter of gene translation [12] or other complex regulatory mechanisms that may be independent of mRNA levels, including phosphorylation pathways influenced by fluid dynamics [22,23,24].

A significant methodological advantage of this protocol is the incorporation of pulsatile flow and precisely calculated ESS intensities derived from human exercise tests. This will allow researchers to tailor physiologically relevant, reproducible, and modifiable in vitro flow profiles. Furthermore, using common molecular biology techniques will ensure compatibility with standard laboratory equipment and expand the scope of potential biomarkers that can be studied beyond those highlighted in this study.

Despite these strengths, some limitations should be highlighted. This study used HUVEC; even though widely accepted, it may not completely represent arterial endothelial cells exposed to higher and more variable ESS in vivo. Future studies could consider validating the protocol using artery-derived endothelial cell lines or a primary culture of arterial endothelial cells. Additionally, this method uses an unchanged ESS intensity rarely maintained in real-life situations due to breaks during physical activity, physiological adjustments, and environmental factors. Therefore, caution should be used when generalizing these in vitro results.

## 5. Conclusions

This protocol provides a practical approach for replicating exercise-induced ESS in vitro and assessing its effects on endothelial cells’ gene and protein expression. By integrating real-time human exercise data with an in vitro model, this protocol addresses a critical gap between clinical exercise physiology and cellular vascular biology. The outcomes showed the feasibility of stimulating exercise-induced ESS in vitro and showed an intensity-dependent response at the gene and protein levels.

In conclusion, these results show the potential of this model for broader applications in vascular biology, cardiovascular research, and exercise physiology. For example, this model could be used to study endothelial responses in cells from patients with cardiovascular risk factors and to evaluate the effects of pharmacological agents in exercise-like flow, or to investigate the effects of different exercise modalities on endothelial mechanotransduction pathways. Future developments and the inclusion of additional molecular markers will strengthen the utility of this method to investigate the molecular changes associated with different exercise intensities, pharmaceutical interventions, or pathologies, ultimately helping the development of targeted therapeutic strategies.

## Figures and Tables

**Table 1:** Specific reagent list, used in cell culture, RT-qPCR, and Western blot.

Reagent	Manufacturer	Catalog No.
MesoEndo Cell Growth Medium	Cell Applications (San Diego, CA, USA)	211-500
Human Umbilical Vein Endothelial Cell (HUVEC)	Cell Applications	200-05n
Ibidi μ-Slide with IbiTreat	Ibidi (Fitchburg, WI, USA)	80166
Yellow-Green Perfusion Set	Ibidi	10964
eNOSp	Fisher Scientific (Hampton, NH, USA)	BP1600-100
Alexa Fluor 488	Invitrogen (Waltham, MA, USA)	A11094
RNeasy Micro Kit	Qiagen (Germantown, MD, USA)	74004
High-Capacity cDNA Reverse Transcription Kit	Applied Biosystems (Waltham, MA, USA)	43-688-14
TaqMan Fast Advanced Master Mix	Applied Biosystems	44-445-56
Cell Lysis Buffer	Cell signaling (Danvers, MA, USA)	9803
4X Protein sample loading buffer	Licor (Lincoln, NE, USA)	928-40004
4–15% Mini-PROTEAN TGX Precast Protein Gels	Biorad (Hercules, CA, USA)	4561083
Precision Plus Protein Dual Color Standards	Biorad	1610374
Trans-Blot Turbo Mini PVDF Transfer Packs	Biorad	1704156
Intercept (TBS) Blocking Buffer	Licor	927-60001
eNOS Primary Antibody	Abcam (Waltham, MA, USA)	EPR23750-3
IRDye 800	Licor	926-32210

**Table 2:** Specific equipment required for the determination of endothelial shear stress, RT-PCR, and Western blot.

Equipment	Manufacturer
TrueOne 2400	Parvo Medics (Salt Lake City, UT, USA)
H10 Heart Rate Sensor	Polar (Lake Success, NY, USA)
Lactate Plus Meter	Nova (Waltham, MA, USA)
StepOne Plus PCR Real-Time System	Applied Biosystems
Mini PROTEAN Tetra Cell Electrophoresis System	Biorad
Trans-Blot Turbo Transfer System	Biorad
Odyssey DLX Imager	Licor

**Table 3:** Volumes of reagents needed for one reverse transcription reaction.

Component	Volume (μL)
10X RT Buffer	2.0
25X dNTP Mix (100 mM)	0.8
10X RT Random Primers	2.0
Multiscribe Reverse Transcriptase	1.0
Nuclease-free Water	4.2
Total for One Reaction	10.0

**Table 4:** Thermocycler set up temperatures and times for cDNA synthesis.

Segment Number	Number of Cycles	Temperature (°C)	Time
1	1	25	10 min
2	1	37	120 min
3	1	85	5 min
4	1	4	∞

**Table 5:** Volumes of reagents needed for one RT-PCR.

Component	Volume (μL)
2X TaqMan Fast Advanced Master Mix	10
20X TaqMan Assay	1.0
Nuclease-free Water	7.0
Total for One Reaction	18.0

**Table 6:** Thermocycler set up temperatures and times for RT-PCR.

Segment Number	Number of Cycles	Temperature (°C)	Time
1	1	50	2 min
2	1	90	20 s
3	40	95	1 s
		60	20 s

## Data Availability

Uncropped Western blot images and the data set can be accessed in the GitHub public repository at https://github.com/daconde7/Quantificaton-of-Genes-and-Proteins-Associated-with-Endothelial-Cell-Function.git (accessed on 8 July 2025).

## Supplementary Materials

The following supporting information can be downloaded at: https://www.mdpi.com/article/10.3390/biology14091189/s1, Figures S1–S3: Western blot image showing eNOS expression with the corresponding loading control GAPDH can be accessed at https://github.com/daconde7/Quantificaton-of-Genes-and-Proteins-Associated-with-Endothelial-Cell-Function.git (accessed on 8 July 2025).
