# Supplementary figures and images for: Quantification of Genes and Proteins Associated with Endothelial Cell Function After Different Exercise-Induced Shear Stress Intensities In Vitro
Source: Biology (Basel). 2025 Sep 3;14(9):1189. doi: 10.3390/biology14091189 (PMC12466968; doi:10.3390/biology14091189)

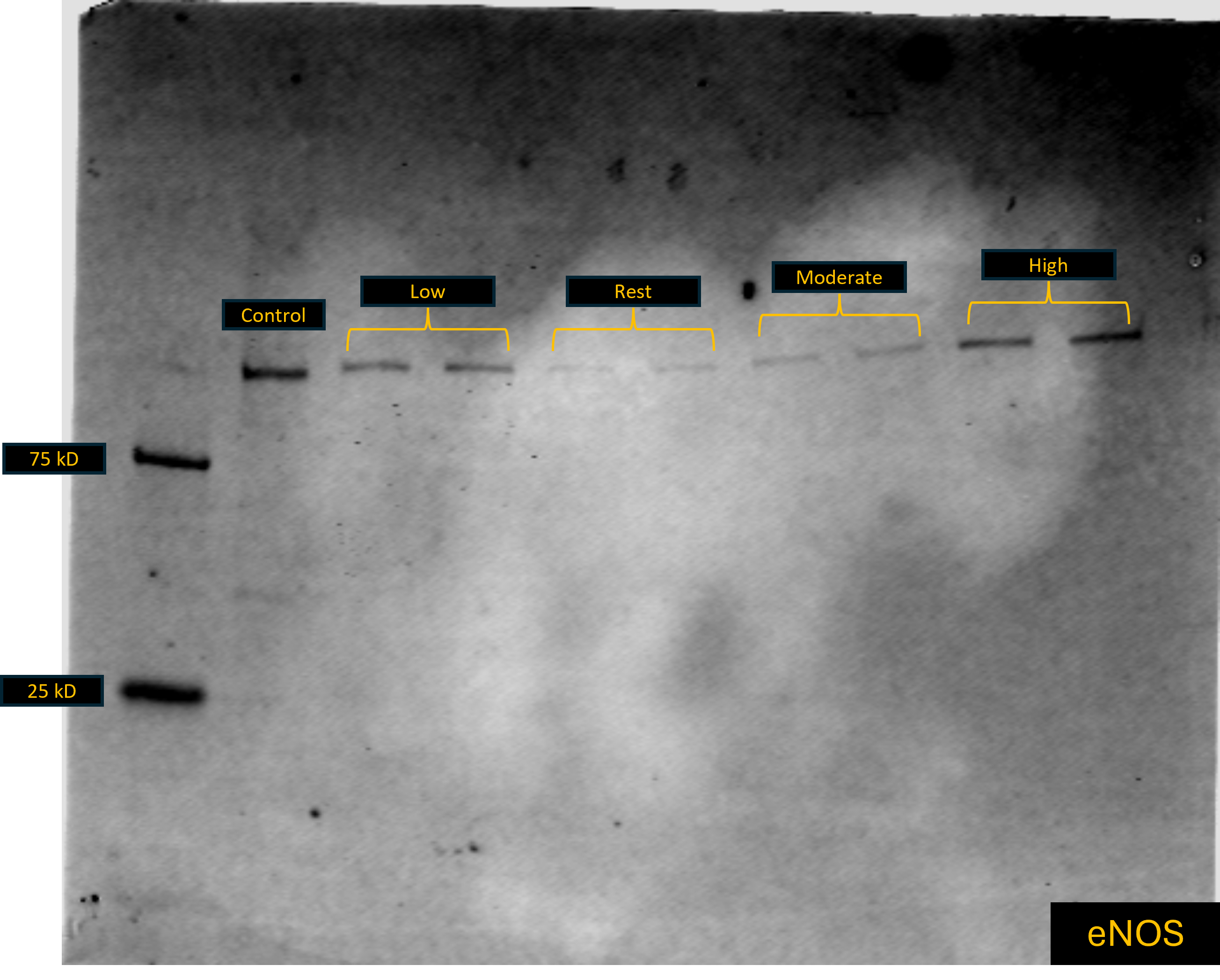

Supplement: Supplementary file 1 [file biology-14-01189-s001.zip › biology-3776745/Figure S1.png]

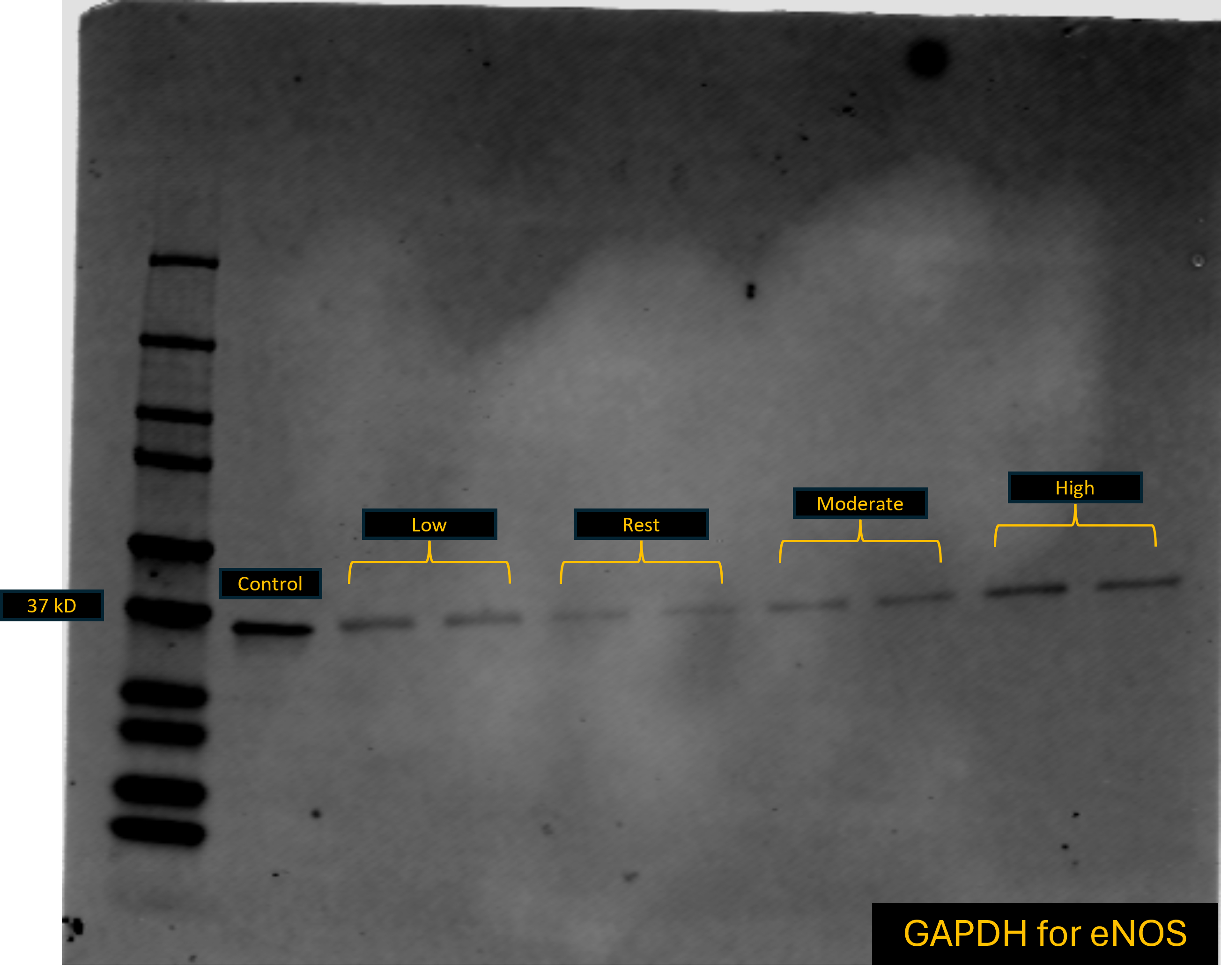

Supplement: Supplementary file 1 [file biology-14-01189-s001.zip › biology-3776745/Figure S2.png]

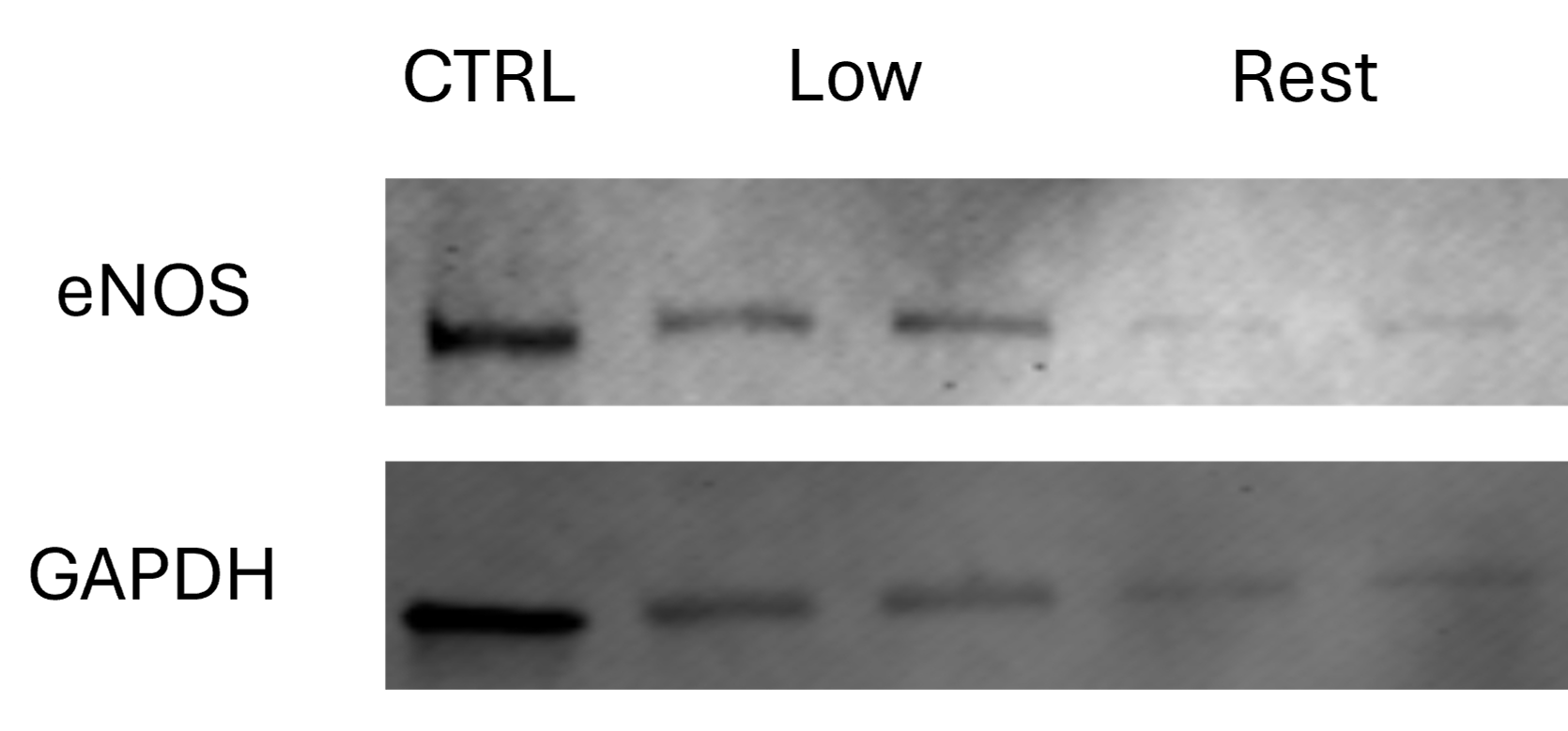

Supplement: Supplementary file 1 [file biology-14-01189-s001.zip › biology-3776745/Figure S3.png]
